# Outcomes of the first adolescent-focused smoking cessation clinic in Türkiye on tobacco control

**DOI:** 10.55730/1300-0144.6114

**Published:** 2025-11-11

**Authors:** Demet TAŞ, Furkan KALAYCI, Alkım ÖDEN AKMAN

**Affiliations:** 1 Divisions of Adolescent Medicine, Department of Pediatrics, Ankara Yıldırım Beyazıt University, Faculty of Medicine, Ankara Bilkent City Hospital, Ankara, Turkiye; 2 Department of Pediatrics, Ankara Bilkent City Hospital, Ankara, Turkiye; 3 Divisions of Adolescent Medicine, Department of Pediatrics, University of Health Sciences, Faculty of Medicine, Ankara Bilkent City Hospital, Ankara, Turkiye

**Keywords:** Adolescent, smoking cessation clinic, pocket money, nicotine patches, quit smoking

## Abstract

**Background/aim:**

Adolescent smoking remains common, highlighting the need to control tobacco use and identify influencing factors. This study aims to evaluate the outcomes of an adolescent-focused smoking cessation clinic and the factors influencing them.

**Materials and methods:**

The study was conducted with 262 adolescents, who, along with their parents, had direct access to the clinic. Data were drawn from patient records containing structured registration and follow-up documentation. The study investigated the effects of baseline cigarette consumption, sociodemographic characteristics, and nicotine patch use on smoking cessation rates at three months, six months, and one year.

**Results:**

The three-month, six-month, and one-year quit rates for adolescents were 18%, 14.3%, and 13.5%, respectively. The regression model revealed a positive association with three-month smoking cessation among those who had a cough (OR = 8.57; 95% CI: 1.83–40.11; p = 0.06) and those who used a nicotine patch (OR = 5.74; 95% CI: 2.06–15.94; p < 0.001). Additionally, individuals who used their pocket money to purchase cigarettes were more likely to quit smoking for three months (OR = 3.47; p = 0.038) and six months (OR = 9.26; p = 0.039). For every 10-cigarette increase in daily consumption, the likelihood of smoking cessation decreased at both 6 months (OR = 0.31; 95% CI: 1.28–8.33; p = 0.014) and one year (OR = 0.26; 95% CI: 1.39–10.42; p = 0.009).

**Conclusion:**

Our findings suggest that not receiving extra pocket money and smoking fewer cigarettes at the time of admission are associated with higher quit rates. Even short-term use of nicotine patches and addressing symptoms such as coughing may support adolescents’ intention to quit. Adolescent smoking cessation clinics provide a valuable opportunity to address these factors in collaboration with families.

## 1. Introduction

Tobacco use is one of the leading preventable causes of death worldwide. Ninety percent of adult tobacco users initiate tobacco use or develop addiction before the age of 18 [1].

Preventing tobacco use is at the forefront of tobacco control, and many school- and community-based programs exist for this purpose. Nevertheless, smoking prevalence has not only stagnated. Still, it has also escalated in particular middle- and low-income countries, and in regions of Eastern Europe, South Asia, and East Asia [2].

In the evaluation of the 15–16 age cohort within the European School Survey Project on Alcohol and Other Drugs (2019 ESPAD), it was determined that 41% had smoked at least once in their lifetime, 20% were recent smokers, and 10% had smoked daily in the preceding 30 days [3]. According to the Global Youth Tobacco Survey in Turkiye, around 10% of adolescents aged 13 to 15 smoke about half a pack of cigarettes per day. This rate rises to 30% among individuals aged 15 or older [4]. As seen in studies, the smoking rate among adolescents is quite high, and these adolescents need intervention and smoking prevention.

Monitoring of adolescent smokers revealed that their rates of spontaneous smoking cessation were notably low, like those of adults. A longitudinal study found that annual smoking cessation rates during the transition from adolescence to young adulthood ranged from 2.3% to 4.1% [5]. Research indicates that more than half of teenagers express their desire to quit smoking when asked. Since the 1990s, there has been a shift away from the belief that adolescents who smoke will automatically quit. In many countries, various social, educational, or health intervention programs have been established to help adolescents quit smoking. Sussman et al. found that various school-based intervention groups were about twice as likely to quit smoking as control groups. In addition, it is recommended that all physicians provide smoking cessation interventions for adolescents [6–8].

The literature rarely reports on adolescent-focused smoking cessation clinics that adolescents or their families can directly visit. Such clinics could enhance smoking cessation rates among adolescents by evaluating their sociodemographic characteristics, identifying smoking-related symptoms that may not be explicitly reported, and providing individualized counseling.

This study was based on the hypothesis that smoking behavior at admission and the use of nicotine patches among adolescents influence one-year smoking cessation outcomes. The aim of this study was to evaluate smoking cessation rates at three months, six months, and one year, and to identify factors influencing smoking cessation among adolescents attending the first adolescent smoking cessation clinic in our country.

## 2. Materials and methods

### 2.1. Study design and population

In this study, we used data from an outpatient clinic for adolescent smoking cessation at a children’s hospital between June 2021 and December 2022. This was a retrospective cross-sectional study, and participants did not receive any form of compensation. Adolescents aged 12 to 18 can access the outpatient clinic for smoking cessation through the medical appointment system or by referral from other pediatric services. The adolescent’s enrollment form and the hospital’s digital data system provided the data for this study. All adolescents and parents attending the clinic were asked to provide consent, including to be called back for follow-up.

Two pediatricians, one psychologist, and a nurse experienced in adolescent health provide services at an adolescent smoking cessation clinic. During the initial visit, adolescents completed forms containing sociodemographic information. At admission, the psychologist assessed the adolescent’s motivation to quit and screened for psychiatric conditions that were exclusion criteria for the study (e.g., schizophrenia, major depression, suicide attempts, intellectual disabilities). Follow-up sessions included motivational interviewing and support for managing withdrawal symptoms, tailored to the adolescent’s readiness to quit. No specific smoking cessation model was applied in this study. Individuals with a history of at least one visit to a psychiatrist or psychologist were classified as having potential mild mental health conditions. The study included adolescents who attended at least one intervention session after enrolling in the adolescent smoking cessation clinic.

Adolescents were regularly invited to the clinic. For those who missed follow-up visits, both the adolescent and their caregiver were contacted separately by phone within the same week to assess recent smoking behavior.

### 2.2. Data included in the study

The following demographic data were included in the study: age, age at smoking initiation, sex (female or male), average number of cigarettes currently smoked per day, and smoking pack-year ([average number of cigarettes smoked per day ÷ 20] × total number of years smoked). Pack-year is a calculation of the total number of cigarettes smoked to date, providing information on the adolescent’s cumulative cigarette consumption throughout their life.

The modified Fagerstrom (mFg) scale was used to measure nicotine dependence in adolescents. MFg is a nicotine dependence scale consisting of seven multiple-choice questions. It is scored based on the number of items checked, with scores ranging from 0 to 9. Higher scores indicate stronger dependence; details are available in the literature [9].

The following information, provided by the adolescents, was taken from the enrollment forms: who the adolescent smoked their first cigarette with (a peer, another person, or alone); how they obtained money for cigarettes (using their own pocket money, get extra pocket money, or through earned income); educational status (currently in formal education, dropped out, or working); which family members smoked (neither parent, only mother, only father, or both); and which family members were aware of the adolescent’s smoking (only the father was unaware, or everyone was aware). Although no complaints were reported spontaneously, department physicians asked both adolescents and their parents about cough symptoms and whether the adolescent had experienced any difficulty performing moderate physical activities, such as going up stairs or walking uphill. The article identifies this difficulty as a limitation in physical activity.

We recommended nicotine replacement therapy (NRT), such as a nicotine patch and/or gum, depending on the number of cigarettes smoked and the adolescent’s mFg score [10]. Adolescents had free access to nicotine patches but had to pay for nicotine gum. Adolescents who were recommended NRT were asked whether they were using NRT as recommended (complying with), how many weeks they were using it, and about any side effects. Information about side effects included the following: itching/redness, pain/burning in the arm, and other (open-ended) side effects for nicotine patches. We asked participants about nausea, taste aversion, and other (open-ended) issues related to nicotine gum.

We assessed the adolescents’ smoking cessation at three months, six months, and one year, commencing from the second week of the first smoking intervention. We also evaluated the average number of cigarettes smoked per day in the first, second, and fourth quarters after admission and whether this number had decreased, ceased, increased, or remained unchanged compared with the number smoked at admission.

### 2.3. Statistical analysis

The data underwent analysis using IBM SPSS Statistics for Windows, Version 26.0 (IBM Corp., Armonk, NY, USA). The normality of the data was assessed using a dual approach that included a visual review and a Shapiro–Wilk test. Normally distributed continuous values were reported as mean and standard deviation (SD), while nonparametric continuous values were reported as median, minimum, and maximum. We reported discrete variables as frequency and percentage. We conducted an independent-samples t-test to compare two groups of parametric variables. Conversely, the nonparametric variables were compared with a Mann–Whitney U test in two independent groups. To assess any differences between discrete groups, the chi-square test or Fisher’s exact test was utilized. Continuous variables between three or more groups were compared using one-way analysis of variance (ANOVA). If a significant difference in mean values between at least two groups was found in a one-way ANOVA test, a pairwise comparison was conducted using a post hoc test of choice, either Tamhane’s T3 or Bonferroni. We determined factors associated with an outcome (e.g., quitting smoking at the third, sixth, and twelfth months) using a logistic regression model with backward selection and a p threshold of 0.10. We used variables of sex, history of chronic disease, history of mental condition, presence of at least one smoker in the family, cigarette money source, whether family members know the adolescent is smoking or not, cough, number of cigarettes used per day, pack-year, mFg, and regular use of nicotine patch as independent variables in the logistic regression model. We assessed the significance of the independent variables on the dependent variable using odds ratios (ORs) and 95% confidence intervals (CIs). p < 0.05 was accepted as significant.

## 3. Results

The study included 262 adolescents, 85 (32.4%) females (F) and 177 (67.6%) males (M). The mean age of the adolescents was 16.4 ± 1.3 years (F: 16.4 ± 1.2; M: 16.4 ± 1.3), and the mean age of smoking initiation was 12.9 ± 2.2 years. The age at which smoking initiation occurred ranged from 6 to 17 years.

The mean number of cigarette pack-years was 2.2 ± 2 (F: 2.2 ± 2.1; M: 2.2 ± 1.9), the mean number of cigarettes smoked per day was 15.0 ± 8.8 (F: 13.3 ±7.8; M: 15.8 ± 9.1), and the mean mFg was 4.5 ± 1.9 (F: 4.5 ± 2.0; M: 4.6 ± 1.8). While the difference in the number of cigarettes smoked according to sex was significant (p = 0.034), no significant difference was found between pack-years and mFg (p = 0.999 and p = 0.790). The number of cigarettes smoked per day ranged from a minimum of two to a maximum of 50.

Table 1 shows the sociodemographic characteristics and their proportions by sex. A weak negative correlation was found between the number of cigarettes, pack-years, and mFg and the age at which adolescents started smoking (r = − 0.274, p < 0.001; r = − 0.392, p < 0.001; r = − 0.268, p < 0.001).

A total of 76.3% (n = 194) of the adolescents reported that they had smoked their first cigarette with a peer, and 68.7% (n = 180) stated that they had tried to quit smoking before. Cough was found in 49% (n = 128) of the adolescents, and difficulty with physical activity in 50% (n = 130). There was no significant difference in cough or difficulty with physical activity by sex (p = 0.561; p = 0.426, respectively). Among those who quit smoking at three months, six months, and one year, the rates of adolescents with mild mental health problems were 45.7%, 41.8%, and 44.4%, respectively. No significant differences in smoking cessation rates were observed between those with and without mild mental health problems (p = 0.580, p = 0.505, p = 0.744).

All adolescents underwent behavioral therapy, with 76.7% (n = 201) receiving additional recommendations for NRT methods. Among the NRT treatments, only 31.8% (n = 64) of participants used nicotine patches as recommended. The use of nicotine gum was reported to be sporadic, lasting only a few days, and its impact on smoking cessation was not evaluated; however, side effects were assessed. The mean duration of use of the nicotine patch was 2.8 ± 2.1 weeks. Among adolescents who used nicotine patches, two reported fatigue, five reported headaches, five experienced arm pain or a burning sensation, and 17 reported itching at the patch application site. Among those who used nicotine gum but did not provide complete information regarding its duration and frequency of use, one reported dizziness, two tachycardia, 13 a bad taste, and 15 nausea. The smoking cessation rate in the first three months was 18% (46 out of 255 adolescents). Those who used nicotine patches as recommended and for a longer period (weeks) had significantly higher smoking cessation rates at three months compared with individuals who maintained, reduced, or increased their smoking (p < 0.001; p < 0.001). No significant difference was found in three-month smoking cessation according to sex (p = 0.683) (Table 2). The following factors were associated with an increased likelihood of smoking cessation at three months: spending one’s own pocket money on cigarettes (OR = 3.47; 95% CI: 1.08–11.23; p = 0.038), having a cough (OR = 8.57; 95% CI: 1.83–40.11; p = 0.06), and using nicotine patches (OR = 5.74; 95% CI: 2.06–15.94; p < 0.001).

At the end of the sixth month, 230 adolescents with previous data were reached, and 33 had maintained smoking cessation for six months, resulting in a quit rate of 14.3%. At the six-month follow-up, using pocket money to buy cigarettes was associated with a higher likelihood of quitting (OR = 9.26; 95% CI: 1.12–76.39; p = 0.039), whereas an increase of 10 cigarettes in daily consumption was associated with a significantly lower likelihood of quitting (OR = 0.31; 95% CI: 0.12–0.78; p = 0.014). The rate of recommended nicotine patch use was significantly higher among adolescents who quit smoking for six months (73.9%) compared with those who did not (42.1%) (p = 0.006).

At the end of one year, 228 adolescents with previous data were reached, 31 had quit smoking for the entire year, and the one-year quit rate was 13.5% At the one-year follow-up, each 10-cigarette increase in daily consumption was associated with a decreased likelihood of smoking cessation (OR = 0.26; 95% CI: 0.10–0.72; p = 0.009). The rate of adherence to recommended nicotine patch use was significantly higher among adolescents who quit smoking for one year (73.7%) compared with those who did not (44.3%) (p = 0.024).

Figure shows whether the number of cigarettes smoked was reduced, unchanged, increased, or quit in the first, second, and fourth quarters of the one-year follow-up, compared with baseline, among adolescents.

Among the adolescents interviewed in the first quarter, there was no statistically significant difference between the proportions of adolescents who quit smoking and reduced smoking, and those with unchanging and increased smoking with regards to the presence of chronic disease, the presence of psychiatric symptoms, whether they dropped out of formal education, who in the family smoked, who in the family knew that the adolescent smoked, how the adolescent obtained money for cigarettes, the presence of cough, or difficulty in moderate physical activity (respectively: p = 0.918; p = 0.580; p = 0.186; p = 0.180; p = 0.451; p = 0.247; p = 0.144; p = 0.133; p = 0.970).

The difference in the mean number of cigarettes smoked per day between those who quit and those who continued smoking was not significant at three months (p = 0.099), but became significant at six months (p = 0.011) and one year (p = 0.010). The number of cigarettes consumed, pack-year, and mFg scores of adolescents according to the duration of smoking cessation are shown in Table 3. Three-month (M: 19.2%, F: 14.1%, p = 0.387), six-month (M: 14.1%, F: 9.4%, p = 0.325), and one-year (M: 13.6%, F: 8.2%, p = 0.307) smoking cessation rates did not differ significantly by sex.

Table 4 presents the number of cigarettes smoked, pack-year values, and mFg scores for adolescents by individual sociodemographic characteristics.

The number of cigarettes smoked, pack-year, and mFg scores according to the sociodemographic characteristics of the adolescents, including their parents, and the symptoms of the adolescents are shown in Table 5.

## 4. Discussion

Although the outcomes of smoking cessation clinics for adults are well documented, evidence regarding adolescent smoking cessation mainly comes from school-based intervention studies [6,11,12]. According to one review, the average smoking cessation rate between 3 and 12 months was 12%, compared with 7% in the control group, based on the results of various school-based interventions [6]. This is the first study to evaluate smoking cessation rates and the factors influencing them among adolescents attending the first adolescent-focused smoking cessation clinic in our country. We found that the three-month, six-month, and one-year quit rates for adolescents were 18%, 14.3%, and 13.5%, respectively.

A subset of research into smoking cessation among adolescents consists of intervention trials, which primarily evaluate the efficacy of various behavioral therapy models and NRT. In a randomized controlled trial in adolescents, the three-month abstinence rate was 18% with nicotine patches and behavioral therapy, 6.5% with nicotine gum, and 2.5% in the control group. Compliance was higher with the nicotine patch than with nicotine gum [13]. A review reported that the 10-week use of nicotine patches resulted in an average abstinence rate of 28% among adolescents. Compliance was inconsistently reported across studies; however, when data were available, NRT compliance rates ranged from 29% to 85% [14].

Since the rate of nicotine gum use was very low in our study, we could not measure compliance with it. Although our study participants were able to receive nicotine patches free of charge and in sufficient quantities, only 32.3% of them complied with the treatment. It was found that adolescents who used the nicotine patch were more likely to quit smoking for three months. However, this effect was not observed among those who had quit smoking for six months or one year. Nevertheless, those who quit for three months, six months, or one year had higher rates of nicotine patch use than those who did not quit. Adolescents who use nicotine patches, even briefly, may exhibit stronger intention and motivation to quit. Side effects associated with NRT were similar to those reported in the literature [13].

Adolescents who smoke fewer cigarettes tend to have higher success in smoking cessation. [15]. Given this, it is important to understand the factors associated with less smoking. Adolescents who used their own pocket money to purchase cigarettes were found to smoke fewer cigarettes. These adolescents were more frequently observed to quit smoking for periods of three and six months. Indeed, in studies conducted in different European countries, it was found that having more pocket money was associated with greater addiction and smoking more cigarettes per day [16].

We found that adolescents whose parents smoked consumed more cigarettes than those whose mother was the only smoker. In addition, adolescents whose fathers did not know that they smoked were less likely to smoke. Especially in Middle Eastern and Asian cultures like ours, the father is the more dominant parent at home [17]. Perceived fear of the father or disapproval of smoking may lead to fewer cigarettes. Studies suggest that the perception of parental disapproval has a protective effect on adolescent smoking and alcohol dependence [18].

Although the males in our study had higher average daily cigarette consumption and pack-year values compared with females, no significant difference was observed in nicotine dependence levels. Similarly, no significant difference was found in smoking cessation rates between sexes. Participants who had dropped out of school were found to have higher levels of cigarette consumption and nicotine dependence. However, whether they continued formal education did not appear to affect smoking cessation rates. There is an association between the number of cigarettes smoked and tobacco dependence, school dropout, and school absenteeism [19]. The presence of a chronic illness or a history of mild psychiatric conditions was also found to have no significant impact on smoking cessation.

Having a cough was found to have a significant positive effect on smoking cessation during the first three months. However, this effect was not observed at the six-month or one-year follow-ups. This effect may be attributed to adolescents’ tendency to normalize or ignore the symptom over time following their initial visit to the smoking cessation clinic. A study conducted among university students found that they underestimated the effects of smoking on the respiratory system and its potential future consequences. However, existing research has shown that smoking impairs respiratory function tests even in the early stages [20,21].

This study has a few limitations. First, the duration of smoking cessation was assessed based on self-reported statements from adolescents and their parents, without the use of any laboratory methods to confirm smoking abstinence. Secondly, this was a retrospective study, and the study population consisted of adolescents who had access to an adolescent smoking cessation clinic. Therefore, the findings may not be generalizable to the broader adolescent population intending to quit smoking. Additionally, the absence of an attrition analysis comparing participants who dropped out with those who completed the study is a limitation, as it may affect the internal validity of the results.

Finally, another limitation of our study is the lack of access to detailed diagnostic information regarding the mild mental health problems among the included participants.

In conclusion, since smoking fewer cigarettes has been identified as a key predictor of cessation success, providing early-stage counseling to adolescents is critically important. Our findings revealed that adolescents who did not receive extra pocket money for cigarettes were more likely to quit smoking. We consider this a valuable finding. Adolescent smoking cessation clinics also provide an opportunity to collaborate with parents in assessing smoking behavior and the factors influencing quitting. Adolescents may perceive even short-term use of nicotine patches as an indicator of their intention to quit smoking. Inquiring about cough symptoms and emphasizing the adverse health effects of smoking in all adolescent smokers appear to be effective in promoting smoking cessation. We believe that an adolescent-focused smoking cessation clinic, accessible to both adolescents and their parents as required, would address an important need.

## Figures and Tables

**Figure:**
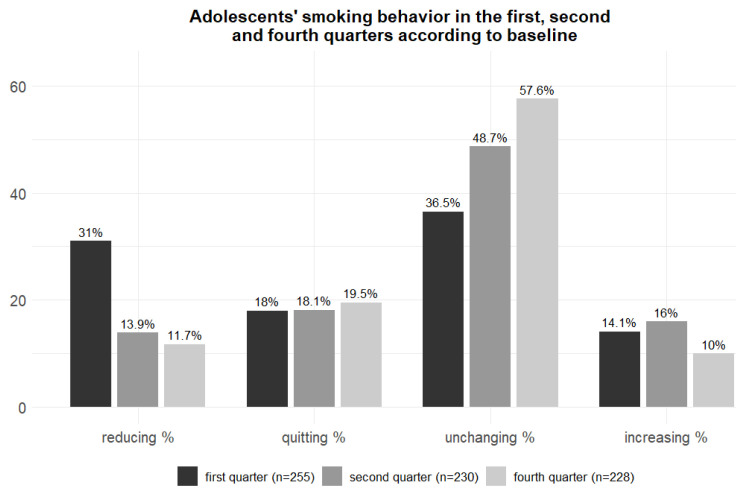
Smoking behaviors in the first quarter, second quarter, and fourth quarter of the follow-up period.

**Table 1:** Demographic and psychosocial characteristics of adolescents by sex.

	Total	Female	Male	p
With chronic disease n (%)	48 (18.3)	18 (21.2)	30 (16.9)	0.264**
Without chronic disease n (%)	214 (81.7)	67 (81.8)	147 (83.1)	
With mental health conditions n (%)	124 (47.3)	54 (63.5)	70 (39.5)	<0.001**
Without mental health conditions n (%)	138 (52.7)	31 (36.5)	107 (60.5)	
Formal education n (%)	204 (78.9)	64 (76.2)	140 (80.2)	0.048***
School dropout n (%)	43 (16.5)	19 (22.6)	24 (13.6)	
Adolescent workers n (%)	12 (4.6)	1 (1.2)	11 (6.2)	
Smoking initiation with a peer n (%)	194 (76.1)	53 (66.3)	141 (80.)	0.001**
Initiation with someone else n (%)	61 (23.9)	27 (33.7)	26 (19.4)	
Buys cigarettes with their own pocket money n (%)	166 (63.4)	61 (71.8)	105 (59.3)	<0.001***
Buys cigarettes with their extra pocket money n (%)	30 (11.5)	15 (17.6)	15 (8.5)	

** Fisher’s exact test,

*** chi-square test, SD: standard deviation

**Table 2:** Adolescents’ smoking behavior for the first three months by sex and NRT use.

Factors affecting smoking behavior		Smoking behavior at third month counselling (n = 255)				p

		Reducing	Quitting	Unchanging	Increasing	

Sex n (%)	Female (n = 82), n (%)	27 (34.2)	12 (26.1)	29 (31.2)	14 (37.8)	0.683*

	Male (n = 173), n (%)	52 (65.8)	34 (73.9)	64 (68.8)	23 (62.2)	

Using nicotine patch n (%)	As recommended (n = 64), n (%)	21 (46.7)	24 (77.4)	13 (31.0)	6 (37.5)	< 0.001*

	Not as recommended (n = 7), n (%)	24 (53.3	7 (22.6)	29 (69.0)	10 (62.5)	

Week of nicotine patch use mean ± SD		2.8 ± 1.8	3.9 ± 2.8^a^	2.1 ± 1.8^b^	1.9 ± 1.0^c^	< 0.001** 0.020^a^–^b^ 0.004^a^–^c^

* chi-square test,

** one-way ANOVA post hoc Tamhane,

SD: standard deviation, NRT: nicotine replacement therapy

**Table 3:** Number of cigarettes per day, pack-year, and mFg score by quit duration.

	Cigarettes/per day		Pack-year		mFg Score	
	Mean ± SD	p	Mean ± SD	p	Mean ± SD	p
Nonsmokers for three months (n = 46)	13 ± 7.5	0.099*	2.1 ± 1.9	0.792*	4.5 ± 1.9	0.829*
Smokers for three months (n = 209)	15.3 ± 8.8		2.2 ± 1.8		4.5 ± 1.8	
Nonsmokers for six months (n = 33)	11.4 ± 6.5	0.011*	1.5 ± 1.1	<0.001*	4.1 ± 1.8	0.116*
Smokers for six months (n = 197)	15.2 ± 9		2.3 ± 2.0		4.6 ± 1.8	
Nonsmokers for one year (n = 31)	11.2 ± 6.5	0.010*	1.5 ± 1.1	0.005*	4.1 ± 11.9	0.166*
Smokers for one year (n = 197)	15.5 ± 8.9		2.2 ± 1.8		4.6 ± 11.8	

* student t-test,

SD: standard deviation

**Table 4:** Number of cigarettes per day, pack-year, and mFg score by sociodemographic characteristics.

	Cigarettes/day		Pack-year		mFg Score	
	Mean ± SD	p	Mean ± SD	p	Mean ± SD	p
With chronic disease	12.6 ± 7.5	0.036^*^	2.2 ± 1.6	0.627^*^	4 ± 1.8	0.023^*^
Without chronic disease	15.5 ± 9.0		2.3 ± 2		4.7 ± 1.9	
Formal education	14.3 ± 8.6^a^	0.005^**^ 0.010^a^–^b^	2 ± 1.7^a^	0.001^**^ 0,029^a^–^b^	4.4 ± 1.8^a^	0.018^**^ 0.030^a^–^b^
School dropout	19.0 ± 9.2^b^		3.2 ± 2.8^b^		5.2 ± 1.8^b^	
Adolescent workers	13.2 ± 8.0		1.8 ± 1.5		3.9 ± 2.1	
Buys with pocket money	13.7 ± 8.1^a^	0.005^**^ 0.033 ^a^–^b^	1.8 ± 1.8^a^	0.001 ^**^ 0.025^a^–^b^ 0.018^a^–^c^	4.3 ± 1.8^a^	0.002^**^ 0.004^a^–^b^
Buys with extra pocket money	17.7 ± 9.1		3 ± 2.1^b^		5.5 ± 1.8 ^b^	
Buys with earned money	17.1 ± 9.7^b^		2.7 ± 2.1^c^		4.8 ± 1.9	

**Table 5:** Number of cigarettes per day and mFg score among adolescents, by parents’ sociodemographic characteristics and adolescents’ symptoms.

	Cigarettes/day		Pack-year		mFg Score	
	Mean ± SD	p	Mean ± SD	p	Mean ± SD	p
No-smoking parents	14.1 ± 7.4	0.046** 0.024^a^–^b^	2.2 ± 1.9	0.981**	4.5 ± 1.8	0.018** 0.021^a^–^b^
Smoking mother	11.6 ± 6.3^a^		2.1 ± 2.1		3.9 ± 1.8^a^	
Smoking father	15.7 ± 9.8		2.3 ± 2.3		4.4 ± 1.9	
Smoking mother and father	16.8 ± 9.4^b^		2.1 ± 2.1		5.2 ± 1.8^b^	
Father unaware teen smokes	12.7 ± 7.3	0.066*	1.4 ± 1.2	<0.001*	4.2 ± 1.8	4.2 ± 1.8	0.193
Everyone knows teen smokes	15.4 ± 9.1		2.4 ± 2.1		4.6 ± 1.9		
With morning cough	16.5 ± 8.9	0.007*	2.5 ± 2.2	0.047*	4.9 ± 1.8	0.003*
No morning cough	13.6 ± 8.5		1.9 ± 1.6		4.2 ± 1.9	
Difficulty in physical activity	17.3 ± 9.3	<0.001*	2.6 ± 2.2	0.001*	5.1 ± 1.8	<0.001*
No difficulty in physical activity	12.8 ± 7.7		1.8 ± 1.5		4 ± 1.8	

* student t-test,

** one-way ANOVA post hoc Tamhane,

SD: standard deviation
